# MUC1-C dictates neuroendocrine lineage specification in pancreatic ductal adenocarcinomas

**DOI:** 10.1093/carcin/bgab097

**Published:** 2021-10-17

**Authors:** Zhou Luan, Yoshihiro Morimoto, Atsushi Fushimi, Nami Yamashita, Wenhao Suo, Atrayee Bhattacharya, Masayuki Hagiwara, Caining Jin, Donald Kufe

**Affiliations:** 1 Dana-Farber Cancer Institute, Harvard Medical School, Boston, MA, USA; 2 Department of Gastroenterology, Tongji Hospital, Tongji Medical College, Huazhong University of Science and Technology, Wuhan, Hubei, China; 3 Division of Molecular Epidemiology, Jikei University School of Medicine, Tokyo, Japan; 4 Department of Pathology, The First Affiliated Hospital, Xiamen University, Xiamen, Fujian, China; 5 Department of Urology, Keio University Medical School, Tokyo, Japan

## Abstract

Pancreatic ductal adenocarcinomas (PDAC) and poorly differentiated pancreatic neuroendocrine (NE) carcinomas are *KRAS* mutant malignancies with a potential common cell of origin. PDAC ductal, but not NE, lineage traits have been associated with cell-intrinsic activation of interferon (IFN) pathways. The present studies demonstrate that the MUC1 C-terminal subunit (MUC1-C), which evolved to protect mammalian epithelia from loss of homeostasis, is aberrantly overexpressed in *KRAS* mutant PDAC tumors and cell lines. We show that MUC1-C is necessary for activation of the type I and II IFN pathways and for expression of the Yamanaka OCT4, SOX2, KLF4 and MYC (OSKM) pluripotency factors. Our results demonstrate that MUC1-C integrates IFN signaling and pluripotency with NE dedifferentiation by forming a complex with MYC and driving the (i) achaete-scute homolog 1 and BRN2/POU3F2 neural, and (ii) NOTCH1/2 stemness transcription factors. Of translational relevance, targeting MUC1-C genetically and pharmacologically in PDAC cells (i) suppresses OSKM, NE dedifferentiation and NOTCH1/2, and (ii) inhibits self-renewal capacity and tumorigenicity. In PDAC tumors, we show that MUC1 significantly associates with activation of IFN signaling, MYC and NOTCH, and that upregulation of the MUC1-C → MYC pathway confers a poor prognosis. These findings indicate that MUC1-C dictates PDAC NE lineage specification and is a potential target for the treatment of recalcitrant pancreatic carcinomas with NE dedifferentiation.

## Teaser

Our results demonstrate that MUC1-C drives PDAC progression by integrating induction of neuroendocrine (NE) dedifferentiation with self-renewal capacity and tumorigenicity, in support of MUC1-C as a druggable target for the treatment of poorly differentiated pancreatic NE carcinomas.

## Introduction

Pancreatic ductal adenocarcinoma (PDAC) is a highly aggressive malignancy with an increasing incidence (1). Curative treatment of PDAC is limited to resection of Stage I/II tumors and there are few therapeutic options for patients with recurrent and unresectable disease, who have a median overall survival of 7–8 months (1,2). PDAC shares certain genetic and clinicopathologic characteristics with poorly differentiated pancreatic neuroendocrine (NE) carcinomas, which may arise from common cells of origin (3–5). Genomic analyses of pancreatic cancer have revealed a mutational landscape with four common oncogenic events in *KRAS*, *TP53*, *SMAD4* and *CDKN2A*, among which *KRAS* mutations are the most frequent being found in up to 90% of tumors (6,7). Two distinct groups of PDAC tumors have been distinguished by the extent of cell-intrinsic interferon (IFN) signaling that is upregulated in ductal-derived *KRAS* mutant cells (8,9). Otherwise, little is known about the effectors that drive lineage plasticity and specification in pancreatic cancer.

The *MUC1* gene appeared in mammals to protect epithelia from the external environment (10). *MUC1* encodes (i) an N-terminal subunit that includes glycosylated tandem repeats and is shed from the cell surface, and (ii) a C-terminal transmembrane subunit (MUC1-C) that is activated by loss of homeostasis and is associated with wound healing responses of inflammation, proliferation and remodeling (10,11). In this way, MUC1-C contributes to epithelial cell plasticity by inducing loss of polarity and the epithelial-mesenchymal transition (10). MUC1-C integrates epithelial-mesenchymal transition with epigenetic reprogramming by activating polycomb repressive complex 2 and DNA methyltransferases, which contribute to the downregulation of tumor suppressor genes (10,12). MUC1-C also induces gene expression by binding directly to transcription factors (TFs), such as MYC, to promote activation of their target genes (10). Other work has demonstrated that MUC1-C regulates gene expression by activating the esBAF and PBAF chromatin remodeling complexes (13–15). These findings have collectively supported a role for MUC1-C in driving lineage plasticity in cancer cell progression (10).

The present work demonstrates that MUC1 is overexpressed in *KRAS* mutant PDAC tumors and in *KRAS* mutant HPAF-II and AsPC-1 PDAC cells. We show that MUC1-C integrates activation of the (i) type I and II IFN pathways, (ii) Yamanaka pluripotency factors (OCT4, SOX2, KLF4, MYC), (iii) the achaete-scute homolog 1 (ASCL1) and BRN2 NE lineage TFs and (iv) the NOTCH1/2 stemness TFs. Our results further demonstrate that MUC1-C integrates induction of NE dedifferentiation with self-renewal capacity and tumorigenicity in PDAC progression, in support of MUC1-C as a druggable target for the treatment of poorly differentiated pancreatic NE carcinomas.

## Materials and methods

### Cell culture

HPAF-II mutant *KRAS* cells (ATCC, Manassas, VA) were cultured in Eagle’s Minimum Essential Medium (ATCC) supplemented with 10% fetal bovine serum. AsPC-1 mutant *KRAS* (ATCC), Panc-1 mutant *KRAS* (ATCC), MiaPaCa-2 mutant *KRAS* (ATCC) and BxPC-3 wild-type *KRAS* (ATCC) cells were cultured in RPMI 1640 medium (Corning, NY) supplemented with 10% fetal bovine serum and 2 mM glutamine. Authentication of the cells was performed by short tandem repeat analysis every 4 months. Cells were monitored for mycoplasma contamination using the MycoAlert Mycoplasma Detection Kit (Lonza, Rockland, ME) every 3 months.

### Gene silencing

MUC1shRNA (MISSION shRNA TRCN0000122938; Sigma), MYCshRNA (MISSION shRNA TRCN0000039642; Sigma) or a control scrambled shRNA (CshRNA; Sigma) was inserted into the pLKO-tet-puro vector (Plasmid #21915; Addgene, Cambridge, MA). The viral vectors were produced in 293T cells as described (16). Cells transduced with the vectors were selected for growth in 1–4 μg/ml puromycin. Cells were treated with 0.1% DMSO as the vehicle control or 500 ng/ml doxycycline (DOX; Millipore Sigma).

### Immunoblot analysis

Total lysates prepared from subconfluent cells were subjected to immunoblot analysis. Immunoblotting was performed with anti-MUC1-C (#16564, 1:1000 dilution; Cell Signaling Technology (CST), Danvers, MA), anti-MYC (#5605, 1:1000 dilution; CST), anti-OCT4 (#2750, 1:1000 dilution; CST), anti-SOX2 (#3579, 1:1000 dilution; CST), anti-KLF4 (#12173, 1:1000 dilution; CST), anti-BRN2 (#12137, 1:1000 dilution; CST), anti-ASCL1 (#GTX129189, 1:2000 dilution; GeneTex, Irvine, CA), anti-SYP (#MA5-16402, 1:200 dilution; Thermo Fisher Scientific, Waltham, MA), anti-AURKA (#ab1287, 1:2000 dilution; Abcam), anti-NOTCH1 (#3608, 1:1000 dilution; CST), anti-NOTCH2 (#5732, 1:1000 dilution; CST) and anti-GAPDH (#5174, 1:2000 dilution; CST).

### RNA-seq analysis

Total RNA from cells cultured separately in triplicates was isolated using Trizol reagent (Invitrogen) as described (16). TruSeq Stranded mRNA (Illumina, San Diego, CA) was used for library preparation. Raw sequencing reads were aligned to the human genome (GRCh38.74) using STAR. Raw feature counts were normalized and differential expression analysis using DESeq2. Differential expression rank order was utilized for subsequent Gene Set Enrichment Analysis, performed using the fgsea (v1.8.0) package in R (16). The TCGA-PAAD/PDAC dataset was obtained from the cBioPortal Cancer Genomic website. Gene sets queried included those from the Hallmark Gene Sets available through the Molecular Signatures Database (16).

### Confocal microscopy

HPAF-II and AsPC-1 cells were fixed in 3.7% paraformaldehyde (Sigma) at room temperature for 15 min. These samples were washed three times with phosphate-buffered saline and then incubated with 0.3% Triton X-100 (Sigma) at room temperature for 10 min. The samples were blocked with 3% BSA and incubated with anti-MUC1-C (#MA5-11202, 1:50 dilution; Thermo Fisher Scientific) or anti-MYC (#5605, 1:500 dilution; CST) at 4°C overnight. The samples were then incubated with goat anti-Armenian hamster IgG H and L labeled with Alexa Fluor 488 (Abcam) and goat anti-rabbit IgG H and L labeled with Alexa Fluor 647 (Abcam) at room temperature for 1 h. DAPI (Sigma) was used for staining of nuclei. The cells were analyzed by confocal microscopy using an inverted Leica TCS SP5 microscope. Immunofluorescence intensities were calculated using ImageJ software.

### Tumorsphere formation assays

HPAF-II/tet-MUC1shRNA and AsPC-1/tet-MUC1shRNA cells (1 × 10^4^) were seeded per well in 24-well ultra-low attachment culture plates (Corning Life Sciences, Corning, NY) in DMEM/F12 50/50 medium (Gibco, Grand Island, NY) with 20 ng/ml epidermal growth factor (Millipore Sigma), 20 ng/ml bFGF (PreproTech, Cranbury, NJ) and 1% B27 supplement (Gibco). Growth factors were replenished every 3 days. Cells were treated with 0.1% DMSO as the vehicle control or 500 ng/ml DOX for 7 days. HPAF-II and AsPC-1 cells (1 × 10^4^) were seeded per well in 24-well ultra-low attachment culture plates in tumorsphere culture medium in the absence or presence of 10 μM GO-203 for 72 h. Tumorspheres with a diameter >50 μm were counted under an inverted microscope in triplicate wells.

### Clonogenic survival assays

Cells were seeded at 1000 cells/well in 6-well plates and treated with (i) vehicle and 500 ng/ml DOX for 6 days, or (ii) 10 μM GO-203 for 4 days. The cells were stained with 0.5% crystal violet in 25% methanol on day 14 after treatment. Colonies >25 cells were counted in triplicate wells.

### Mouse tumor model studies

Six-week-old female nude mice (Taconic Farms, Germantown, NY) were injected subcutaneously in the flank with 2–5 × 10^6^ tumor cells in 100 μl of a 1:1 solution of medium and Matrigel (BD Biosciences). When the mean tumor volume reached 100–150 mm^3^, mice were pair-matched into groups. In studies of HPAF-II/tet-MUC1shRNA and AsPC-1/tet-MUC1shRNA tumors, mice were fed without or with DOX (625 ppm, daily). In studies of HPAF-II tumors, mice were treated intraperitoneally each day with phosphate-buffered saline or GO-203 at a dose of 12 μg/gm body weight. Tumor measurements and body weights were recorded every 3 days. Mice were sacrificed when tumors reached >1000 mm^3^ as calculated by the formula: (width)^2^ × length/2. These studies were conducted in accordance with ethical regulations required for approval by the Dana-Farber Cancer Institute Animal Care and Use Committee under protocol 03-029.

### Statistical analysis

Each experiment was performed at least three times. Data are expressed as the mean ± standard deviation (SD). The unpaired Mann–Whitney *U* test was used to determine differences between means of groups. A *P*-value of <0.05 denoted by an asterisk (*) was considered statistically significant.

## Results

### MUC1-C is upregulated in KRAS mutant PDAC tumors and cell lines

**Figure 1.**
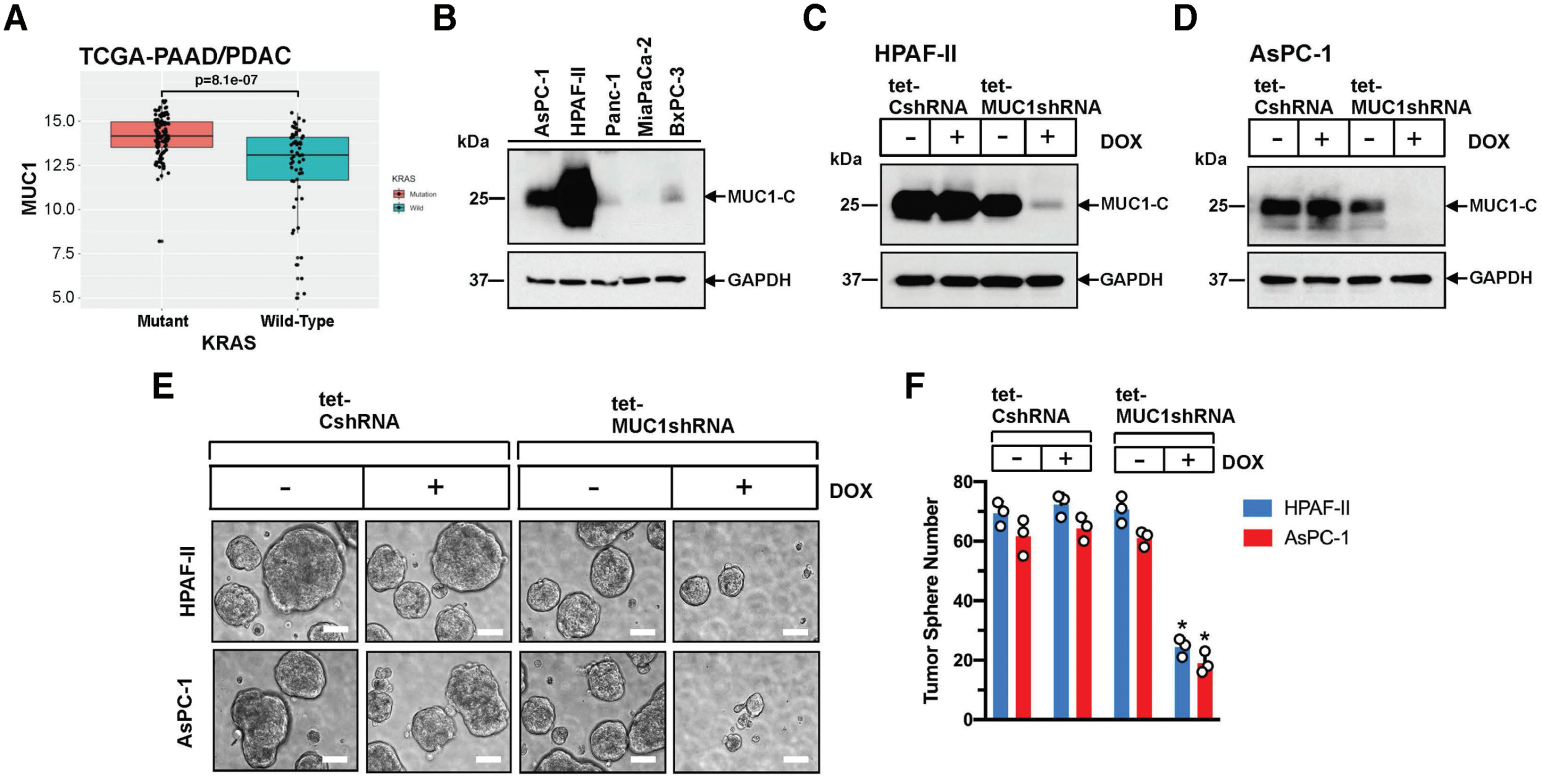
MUC1-C is upregulated in KRAS mutant PDAC cells and is necessary for self-renewal. (**A**) Analysis of the TCGA-PAAD/PDAC dataset comparing MUC1 expression in mutant (*n* = 108) and wild-type (*n* = 55) KRAS PDAC tumors. (**B**) Lysates from the designated PDAC cell lines were immunoblotted with antibodies against the indicated proteins. (**C** and **D**) HPAF-II (**C**) and AsPC-1 (**D**) cells stably expressing a tet-CshRNA or tet-MUC1shRNA were treated with vehicle or 500 ng/ml DOX for 6 days. Lysates were immunoblotted with antibodies against the indicated proteins. (**E**) Representative images are shown for the indicated HPAF-II (upper) and AsPC-1 (lower) cells suspended in tumorsphere medium in the presence of vehicle or DOX for 7 days. (**F**) Number of tumorspheres from three separate determinations of the indicated HPAF-II (blue bars) and AsPC-1 (red bars) cells treated with vehicle or DOX. The asterisk (*) denotes a significant difference from that obtained for DOX-treated tet-CshRNA cells.

Analysis of the 163 PDACs in the TCGA-PAAD dataset, designated TCGA-PAAD/PDAC, demonstrated that (i) *MUC1* is significantly overexpressed in *KRAS* mutant (*n* = 108), as compared to *KRAS* wild-type (*n* = 55), tumors (Figure 1A), including those with different types of *KRAS* mutations (Supplementary Figure S1A, available at *Carcinogenesis* Online). *MUC1* encodes the 158 aa MUC1-C subunit that includes a 58 aa extracellular domain, a 28 aa transmembrane domain and a 72 aa cytoplasmic domain or tail (Supplementary Figure S1B, available at *Carcinogenesis* Online). Analysis of pancreatic cancer cell lines demonstrated that MUC1-C is overexpressed in HPAF-II (KRAS G12D) and AsPC-1 (KRAS G12D) cells (Figure 1B), which were derived from patients with malignant ascites (17–19). By contrast, MUC1-C expression was substantially lower in the Panc-1 (KRAS G12D), MiaPaCa-2 (KRAS G12C) and BxPC-3 (KRAS WT) cell lines established from primary PDACs (20). To address the potential functional significance of MUC1-C expression, we established HPAF-II cells transfected with a tetracycline-inducible control scrambled shRNA (tet-CshRNA) or a MUC1-C shRNA (tet-MUC1shRNA)(Supplementary Figure S1B, available at *Carcinogenesis* Online). Treatment of the HPAF-II transfectants with DOX resulted in the suppression of MUC1-C in HPAF-II/tet-MUC1shRNA and not HPAF-II/tet-CshRNA cells (Figure 1C). As a second model, AsPC-1/tet-MUC1shRNA, but not AsPC-1/tet-CshRNA, cells similarly responded to DOX treatment with downregulation of MUC1-C expression (Figure 1D). HPAF-II and AsPC-1 cells are addicted to mutant KRAS for their survival (21,22). We, therefore, asked if HPAF-II and AsPC-1 cells are dependent on MUC1-C for self-renewal as assessed by the capacity for tumorsphere formation. In this way, HPAF-II and AsPC-1 tumorspheres established in matrigel were serially passaged for assessment of MUC1-C expression (Supplementary Figure S2A, available at *Carcinogenesis* Online). As compared to that found for cells grown as monolayers, MUC1-C levels were clearly upregulated in association with the formation of tumorspheres (Supplementary Figure S2B, available at *Carcinogenesis* Online). Importantly, silencing MUC1-C significantly decreased the number of tumorspheres (Figure 1E and F), demonstrating that these *KRAS* mutant PDAC cells are dependent on MUC1-C for self-renewal.

### MUC1 associates with activation of the type I and II IFN pathways

**Figure 2.**
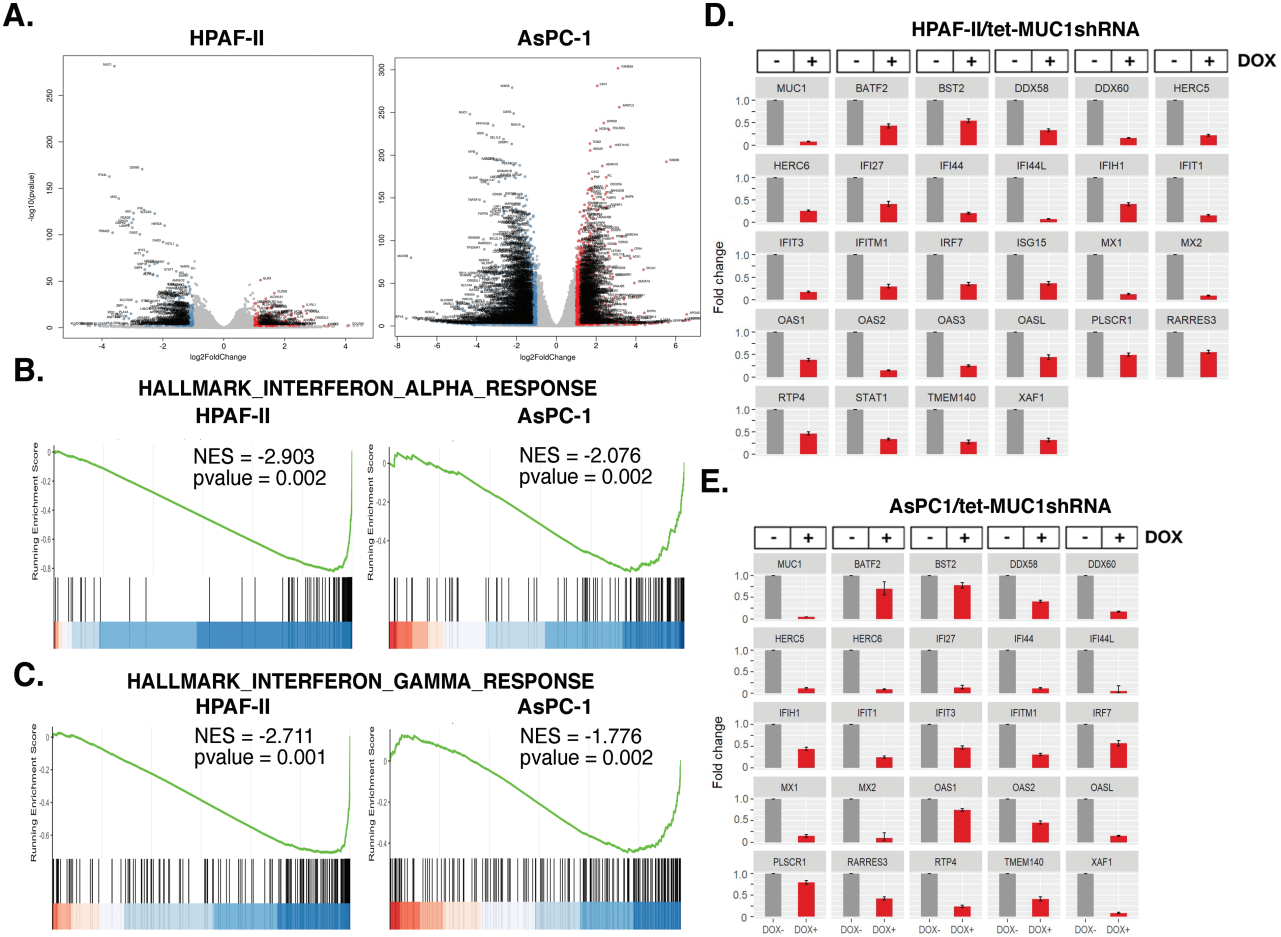
MUC1 associates with activation of the type I and II IFN pathways in HPAF-II and AsPC-1 cells. RNA-seq was performed in triplicate on HPAF-II/tet-MUC1shRNA and AsPC-1/tet-MUC1shRNA cells treated with vehicle or DOX for 6 days. (**A**) Volcano plots of down- and up-downregulated genes in HPAF-II (left) and AsPC-1 (right) cells. (**B** and **C**) The RNA-seq datasets from the HPAF-II (left) and AsPC-1 (right) datasets were analyzed with GSEA using the HALLMARK INTERFERON ALPHA RESPONSE (**B**) and HALLMARK INTERFERON GAMMA RESPONSE (**C**) pathways comparing DOX-treated versus vehicle-treated cells. (**D** and **E**). RNA-seq performed in triplicate on HPAF-II/tet-MUC1shRNA (**D**) and AsPC-1/tet-MUC1shRNA (**E**) cells treated with vehicle (gray bars) or DOX (red bars) for 7 days was analyzed for expression of the indicated ISGs. The results (mean ± SD of three determinations) are expressed as relative mRNA levels compared to that obtained for vehicle-treated cells (assigned a value of 1).

Based on the findings that MUC1-C promotes PDAC cell self-renewal, we performed RNA-seq analyses on triplicate independent cultures of HPAF-II and AsPC-1 cells and found that MUC1-C silencing has global effects on the activation and suppression of their transcriptomes (Figure 2A, left and right). Gene Set Enrichment Analysis of the HPAF-II and AsPC-1 datasets using the HALLMARK collection of gene signatures revealed that MUC1 significantly associates with activation of the HALLMARK INTERFERON ALPHA RESPONSE (Figure 2B, left and right) and HALLMARK INTERFERON GAMMA RESPONSE (Figure 2C, left and right) pathways. Along these lines, *KRAS* mutant PDAC cell-intrinsic activation of an IFN signature has been associated with a ductal-lineage specification (9). Further analysis of the HPAF-II datasets identified MUC1-C-induced genes, such as *STAT1*, which encodes a master regulator of the type I and II IFN pathways (23,24) (Figure 2D). We also found that MUC1-C is necessary for the expression of IFN-stimulated genes (ISGs), including IFIT1/3, OAS1/3, IFITM1, IFI44L and MX1, that promote DNA damage resistance, chronic inflammation and cancer progression (23–27) (Figure 2D). Similar MUC1-C-driven ISGs were identified in AsPC-1 cells (Figure 2E), indicating that MUC1-C integrates activation of IFN pathways and self-renewal in *KRAS* mutant PDAC cell progression. In support of this notion, increasing evidence has linked the innate IFN pathways with stemness (28–31); however, MUC1-C has not been previously implicated in integration of IFN signaling with the cancer stem cell (CSC) state.

### MUC1-C induces the Yamanaka pluripotency factors and NE dedifferentiation

**Figure 3.**
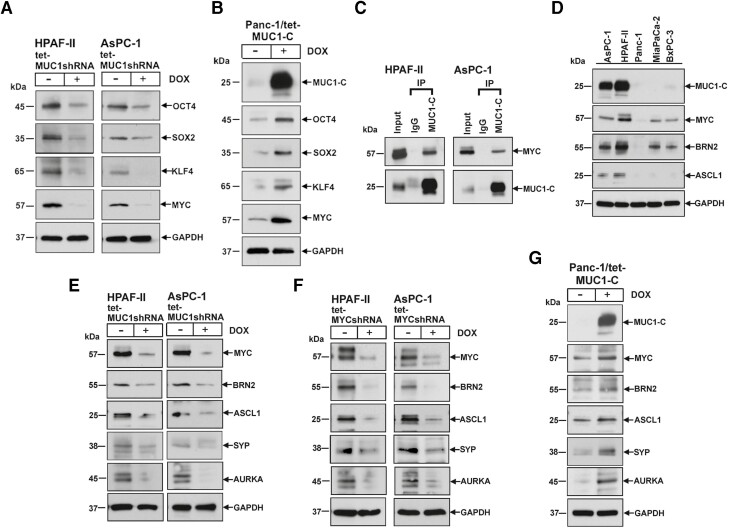
MUC1-C drives expression of the Yamanaka pluripotency factors. (**A**) Lysates from HPAF-II/tet-MUC1shRNA (left) and AsPC-1/tet-MUC1shRNA (right) cells treated with vehicle or DOX were immunoblotted with antibodies against the indicated proteins. (**B**) Lysates from Panc-1 cells expressing a tet-MUC1-C vector treated with vehicle of DOX were immunoblotted with antibodies against the indicated proteins. (**C**) Nuclear lysates from HPAF-II (left) and AsPC-1 (right) cells were precipitated with anti-MUC1-C and a control IgG antibody. Input proteins and the precipitates were immunoblotted with antibodies against MYC and MUC1-C. (**D**) Lysates from the PDAC cell lines were immunoblotted with antibodies against the indicated proteins. (**E**). Lysates from HPAF-II/tet-MUC1shRNA (left) and AsPC-1/tet-MUC1shRNA (right) cells treated with vehicle or DOX were immunoblotted with antibodies against the indicated proteins. (**F**) Lysates from HPAF-II/tet-MYCshRNA (left) and AsPC-1/tet-MYCshRNA (right) cells treated with vehicle or DOX were immunoblotted with antibodies against the indicated proteins. (**G**) Lysates from Panc-1/tet-MUC1-C cells treated with vehicle of DOX were immunoblotted with antibodies against the indicated proteins.

The Yamanaka OCT4, SOX2, KLF4 and MYC (OSKM) pluripotency reprogramming factors confer lineage plasticity and dedifferentiation of fibroblasts (32). Among these, MYC is required for oncogenic *KRAS* signaling and promotes lineage plasticity in PDAC progression (33–36). We found that, like MUC1-C, the OSKM factors are upregulated in HPAF-II and AsPC-1 cells (Supplementary Figure S3A, available at *Carcinogenesis* Online) and that, like MUC1-C, the OSKM factors are induced in AsPC-1 and HPAF-II tumorspheres (Supplementary Figure S3B, left and right, available at *Carcinogenesis* Online), consistent with the involvement of pluripotency in driving stemness and self-renewal (37–39). In concert with these apparent parallels between MUC1-C and OSKM factor expression, silencing MUC1-C in HPAF-II and AsPC-1 cells suppressed OCT4, SOX2, KLF4 and MYC (Figure 3A, left and right). To extend these loss-of-function studies, we transfected Panc-1 cells, which have low levels of MUC1-C expression, with a tet-inducible MUC1-C vector. Treatment of the Panc-1/tet-MUC1-C cells with DOX resulted in the induction of MUC1-C, OCT4, SOX2, KLF4 and MYC (Figure 3B), in support of a MUC1-C → OSKM pluripotency factor pathway. Co-immunoprecipitation studies of nuclear lysates demonstrated that MUC1-C associates with MYC in HPAF-II and AsPC-1 cells (Figure 3C). Confocal microscopy further showed that MUC1-C and MYC colocalize in the nucleus and that, as a control, silencing MUC1-C significantly suppresses nuclear MUC1-C and MYC expression in HPAF-II (Supplementary Figure S4A and B, available at *Carcinogenesis* Online) and AsPC-1 (Supplementary Figure S4C and D, available at *Carcinogenesis* Online) cells. SYP, SNAP25 and chromogranin A are vesicle markers of neuronal cells (36), whereas the master neural BRN2 and ASCL1 TFs drive NE differentiation (40,41). Surprisingly, analysis of HPAF-II and AsPC-1 cells, which have not been previously recognized as having NE features, demonstrated upregulation of BRN2 and ASCL1 (Figure 3D). MUC1-C activates the MYC → BRN2 pathway (16,40). In this regard, expression of MYC and BRN2, as well as ASCL1, were at low to undetectable levels in MiaPaCa-2 and BxPC-3, but upregulated in HPAF-II and AsPC-1, cells (Figure 3D). Along these lines, silencing MUC1-C suppressed expression of MYC, BRN2 and ASCL1 (Figure 3E, left and right). MUC1-C was also necessary for expression of the SYP NE marker and AURKA (Figure 3E, left and right), which has been linked to cell cycle progression of cells with NE dedifferentiation (40,41). As confirmation of the MUC1-C → MYC pathway, silencing MYC also decreased BRN2, ASCL1, SYP and AURKA expression (Figure 3F, left and right). Moreover, treatment of the Panc-1/tet-MUC1-C cells with DOX resulted in the induction of MUC1-C, MYC, ASCL1, BRN2, SYP and AURKA (Figure 3G), in further support of a MUC1-C → MYC NE dedifferentiation pathway.

### Targeting MUC1-C with the GO-203 inhibitor suppresses NE dedifferentiation

Targeting MUC1-C with the GO-203 inhibitor, which blocks MUC1-C homodimerization and nuclear localization (42–44), phenocopies the effects of MUC1-C silencing (16). Inhibiting MUC1-C with GO-203 significantly attenuated nuclear colocalization of MUC1-C and MYC (Supplementary Figure S5A and B, available at *Carcinogenesis* Online). Consistent with the effects of silencing MUC1-C genetically, treatment of HPAF-II and AsPC-1 cells with GO-203 resulted in the downregulation of MUC1-C and suppression of OCT4, SOX2, KLF4 and MYC (Supplementary Figure S5C, left and right, available at *Carcinogenesis* Online). In addition, we found that GO-203 suppresses ASCL1, BRN2 and AURKA in HPAF-II and AsPC-1 cells (Supplementary Figure S5D, left and right, available at *Carcinogenesis* Online). In further support of driving dedifferentiation, targeting MUC1-C with GO-203 decreased the capacity of HPAF-II and AsPC-1 CSCs to form tumorspheres (Supplementary Figure S5E, left and right, available at *Carcinogenesis* Online).

### MUC1-C integrates NE dedifferentiation with induction of NOTCH1/2 expression

**Figure 4.**
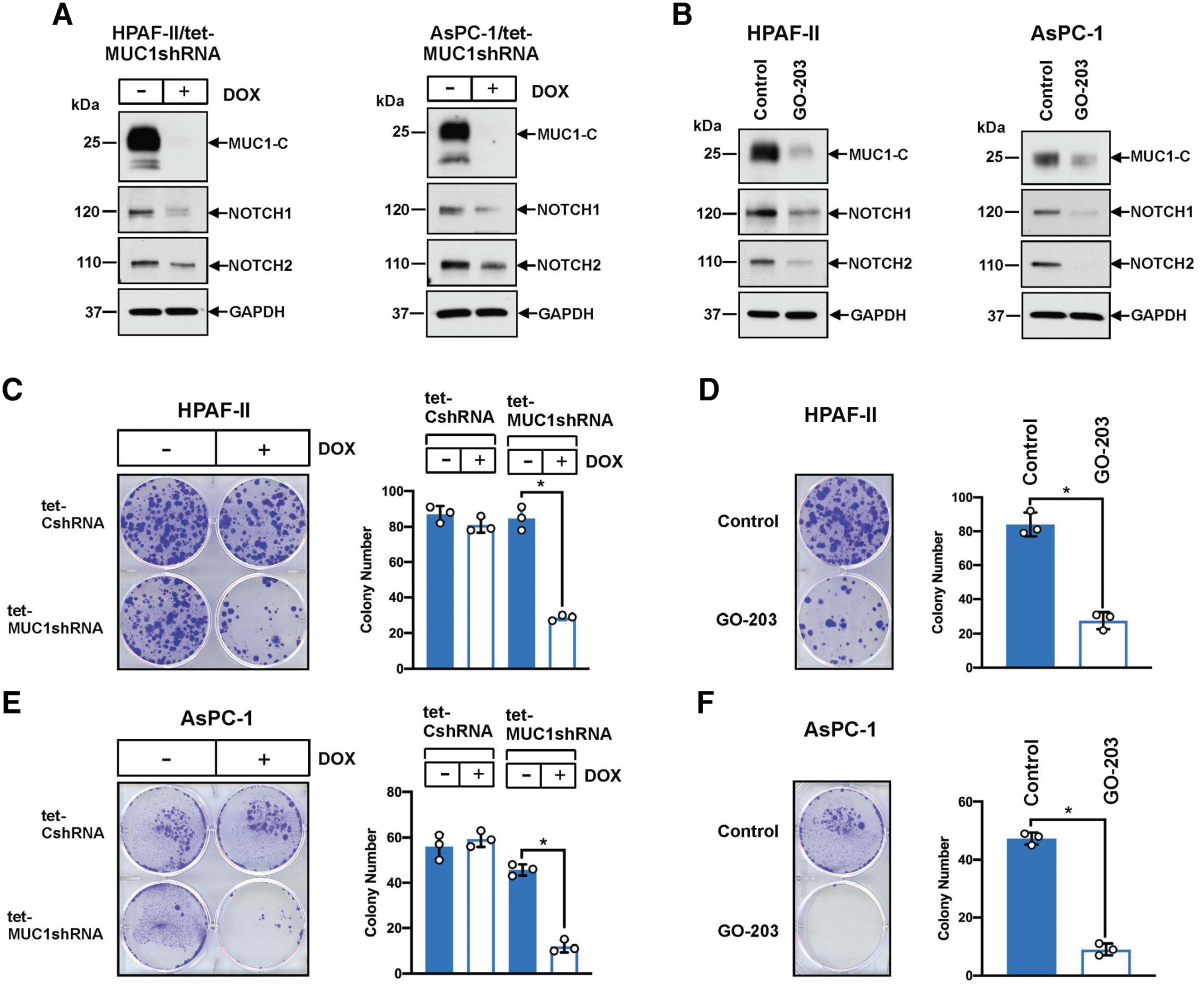
Targeting MUC1-C suppresses NOTCH expression and clonogenicity. (**A**) Lysates from HPAF-II/tet-MUC1shRNA (left) and AsPC-1/tet-MUC1shRNA (right) cells treated with vehicle or DOX for 6 days were immunoblotted with antibodies against the indicated proteins. (**B**) Lysates from HPAF-II (left) and AsPC-1 (right) cells left untreated or treated with 10 μM GO-203 for 48 h were immunoblotted with antibodies against the indicated proteins. (**C**) The indicated HPAF-II cells treated with vehicle or DOX for 6 days were assayed for colony formation. (**D**) HPAF-II cells left untreated or treated with 10 μM GO-203 for 4 days were assayed for colony formation. Colonies were stained with crystal violet on day 14 after treatment (left). Colony number is expressed as the mean ± SD of three independent replicates (right). (**E**) The indicated AsPC-1 cells treated with vehicle or DOX for 6 days were assayed for colony formation. (**F**) AsPC-1 cells left untreated or treated with 10 μM GO-203 for 4 days were assayed for colony formation. Colonies were stained with crystal violet on day 14 after treatment (left). Colony number is expressed as the mean ± SD of three independent replicates (right).

NOTCH1 signaling has been linked to driving NE dedifferentiation of CSCs (14). Additionally, NOTCH2 is a marker of NE stem cells, which initiate NE reprogramming after injury and are the proposed origin of small cell lung cancer (45). In light of the importance of NOTCH signaling in NE dedifferentiation, we found that silencing MUC1-C suppresses NOTCH1/2 in HPAF-II (Figure 4A, left) and AsPC-1 (Figure 4A, right) cells. In addition, targeting MUC1-C with GO-203 decreased NOTCH1/2 expression (Figure 4B, left and right). In extending these results to functional studies, silencing MUC1-C or targeting MUC1-C with GO-203 inhibited HPAF-II CSC clonogenic survival (Figure 4C and D, left and right). Taken together with similar results in AsPC-1 cells (Figure 4E and F, left and right), these findings supported involvement of MUC1-C in integrating the induction of IFN signaling pathways and pluripotency factors with NOTCH1/2 expression in driving NE dedifferentiation.

### MUC1-C is necessary for HPAF-II and AsPC-1 tumorigenicity

**Figure 5.**
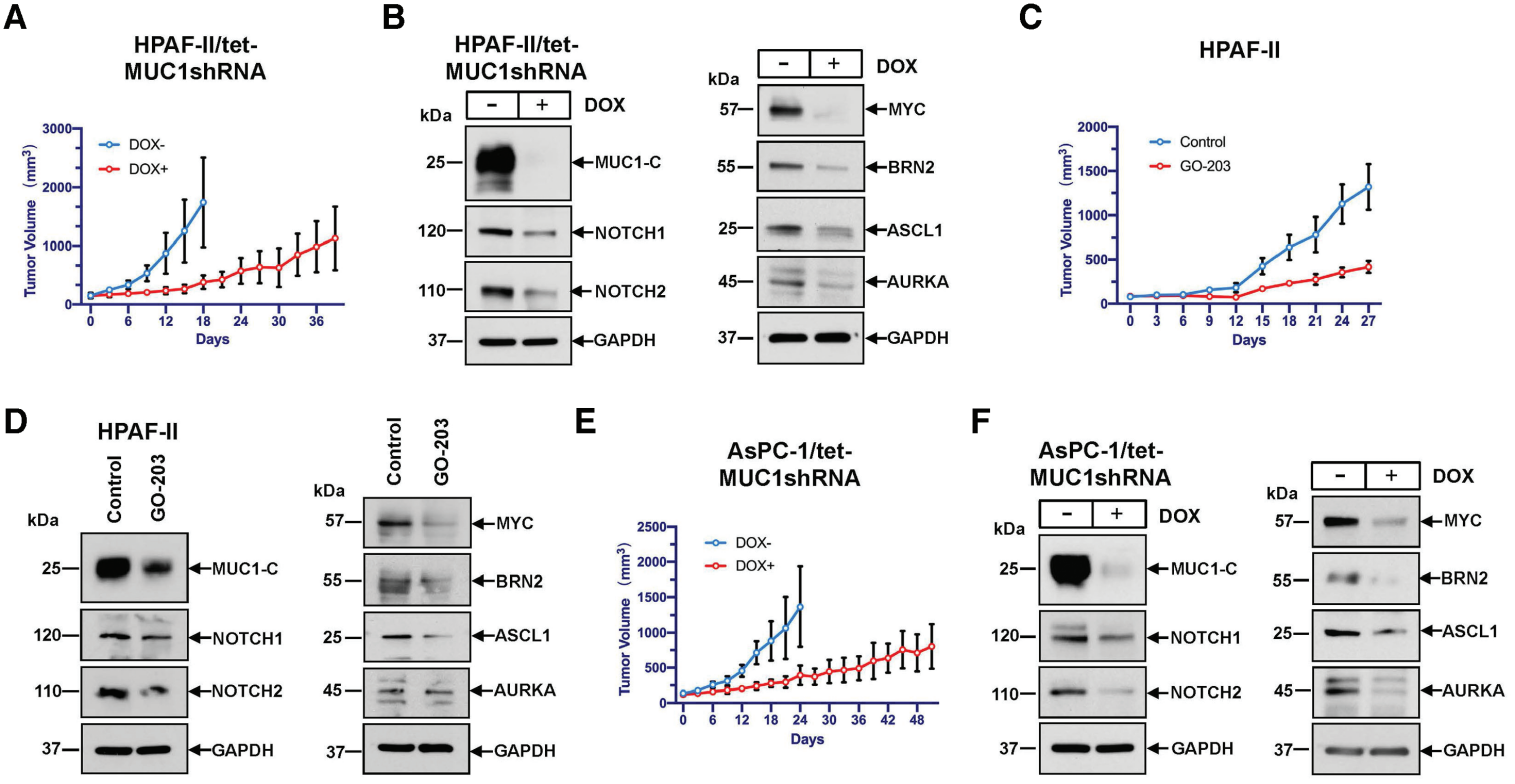
Targeting MUC1-C inhibits HPAF-II and AsPC-1 tumorigenicity. (A) Six-week-old female nude mice were injected subcutaneously in the flank with 3.5 × 10^6^ HPAF-II/tet-MUC1shRNA cells. Mice were pair-matched into two groups when tumors reached approximately 150 mm^3^ and were fed without and with DOX. Tumor volumes are expressed as the mean ± SD for six mice. (**B**) Lysates from HPAF-II/tet-MUC1shRNA tumors obtained on day 18 were immunoblotted with antibodies against the indicated proteins. (**C**) Six-week-old female nude mice were injected subcutaneously in the flank with 2 × 10^6^ HPAF-II cells. Mice were pair-matched into two groups when tumors reached approximately 100 mm^3^ and were treated intraperitoneally each day with phosphate-buffered saline or GO-203 at a dose of 12 μg/gm body weight. Tumor volumes are expressed as the mean ± SD for four mice. (**D**) Lysates from control and GO-203-treated tumors obtained on day 28 were immunoblotted with antibodies against the indicated proteins. (**E**) Six-week-old female nude mice were injected subcutaneously in the flank with 3.5 × 10^6^ AsPC-1/tet-MUC1shRNA cells. Mice were pair-matched into two groups when tumors reached approximately 150 mm^3^ and were fed without and with DOX. Tumor volumes are expressed as the mean ± SD for six mice. (**F**) Lysates from AsPC-1/tet-MUC1shRNA tumors obtained on day 24 were immunoblotted with antibodies against the indicated proteins.

In determining whether MUC1-C drives tumorigenicity, we established HPAF-II/tet-MUC1shRNA tumor xenografts in nude mice and found that feeding DOX to suppress MUC1-C expression significantly decreased tumor growth (Figure 5A). In addition, analysis of lysates from control and DOX-treated HPAF-II tumors confirmed that silencing MUC1-C results in the downregulation of NOTCH1/2 (Figure 5B, left), as well as MYC, ASCL1, BRN2 and AURKA (Figure 5B, right). These results were supported by similar effects of treating mice harboring established (i) HPAF-II tumors with GO-203 (Figure 5C and D, left and right) and (ii) AsPC-1/tet-MUC1shRNA xenografts with DOX (Figure 5E and F, left and right).

### MUC1 associates with activation of the MYC pathway in conferring a poor prognosis

**Figure 6.**
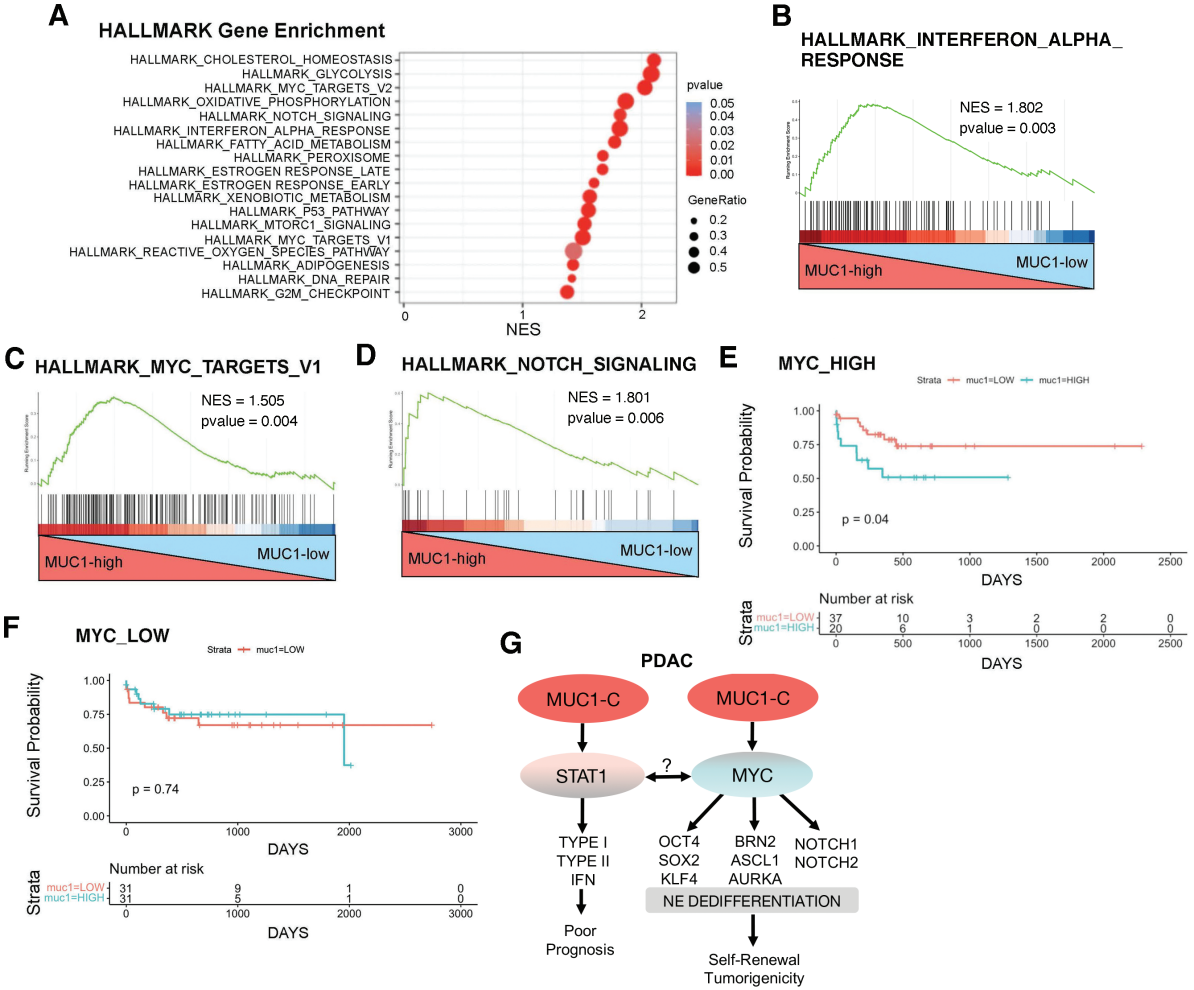
MUC1 associates with MYC and decreased survival in the TCGA-PAAD dataset. (A) GSEA of the 163 PDAC tumors in the TCGA-PAAD/PDAC dataset for associations of MUC1 expression with the indicated HALLMARK gene signatures. (B–D) Enrichment plots for the HALLMARK INTERFERON ALPHA RESPONSE (**B**) HALLMARK MYC TARGETS V1 (**C**) and HALLMARK NOTCH SIGNALING (**D**) pathways, comparing MUC1-high to MUC1-low PDAC tumors in the TCGA-PAAD/PDAC dataset. (**E**) Probability of survival comparing MUC1-high, MYC-high (blue curve) to MUC1-low, MYC-high (red curve) PDAC tumors in the TCGA-PAAD/PDAC dataset. (**F**) Probability of survival comparing MUC1-high, MYC-low (blue curve) to MUC1-low, MYC-low (red curve) PDAC tumors in the TCGA-PAAD/PDAC dataset. (**G**) Schematic representation of MUC1-C in driving PDAC NE lineage specification. MUC1-C induces STAT1 and the type I and II IFN pathways, which are associated with *KRAS* mutant PDAC tumors and poor patient outcomes (9). MUC1-C also activates MYC, which is required for oncogenic mutant *KRAS* signaling in PDACs. Further studies are needed, at least in part, to determine whether MUC1-C independently activates STAT1 and MYC. The present data further indicate that MUC1-C integrates MYC activation with induction of the (i) OCT4, SOX2 and KLF4 pluripotency factors, (ii) BRN2, ASCL1 and AURKA NE markers, and (iii) NOTCH1/2 stemness TFs. In this way, MUC1-C promotes NE dedifferentiation, self-renewal capacity and tumorigenicity in PDAC progression.

In extending these results, analysis of the 163 PDACs in the TCGA-PAAD dataset demonstrated that MUC1-high tumors significantly associate with activation the HALLMARK INTERFERON ALPHA RESPONSE signature (Figure 6A and B), consistent with findings in HPAF-II and AsPC-1 cells. In addition, MUC1-high tumors were significantly associated with activation of the HALLMARK MYC TARGETS V1 and V2 (Figure 6C; Supplementary Figure S6A, available at *Carcinogenesis* Online) and the HALLMARK NOTCH SIGNALING (Figure 6D) pathways. MUC1 also significantly correlated with AURKA (Supplementary Figure S6B, available at *Carcinogenesis* Online) and NOTCH2 (Supplementary Figure S6C, available at *Carcinogenesis* Online) expression. We found that MUC1-high/MYC-high, as compared to MUC1-low/MYC-high, tumors associate with significant decreases in patient survival (Figure 6E). In contrast, there was no significant effect on survival of patients with MUC1-high/MYC-low and MUC1-low/MYC-low tumors (Figure 6F), in concert with adverse clinical outcomes in association with activation of the MUC1-C → MYC signaling pathway.

## Discussion

PDACs are associated with *KRAS*, *CDKN2A*, *TP53* and *SMAD4* mutations (6,7). PDACs with NE features are poorly differentiated and harbor *KRAS* and *TP53*, as well as *RB1*, mutations (4,5). We found that MUC1 is significantly overexpressed in PDAC tumors that harbor *KRAS* mutations. We also found that MUC1-C is overexpressed in *KRAS* mutant HPAF-II and AsPC-1 cell lines that were derived from patients with advanced PDAC. Accordingly, we silenced MUC1-C in these cells and, interestingly, found by RNA-seq analysis highly significant associations of MUC1 with the HALLMARK INTERFERON ALPHA RESPONSE and HALLMARK INTERFERON GAMMA RESPONSE pathways (Figure 6G). MUC1 expression was also significantly associated with activation of the HALLMARK INTERFERON ALPHA RESPONSE pathway in PDAC tumors. MUC1-C binds directly to STAT1 and promotes the activation of STAT1 target genes (46). Our results demonstrate that MUC1-C is necessary for STAT1 expression and induction of the type I and II IFN pathways in PDAC cells (Figure 6G). Activation of type I and II IFN signaling has been associated with progression of the CSC state (28,29,31) and, in PDAC tumors, to aggressive disease and a poor prognosis (9). Notably, MUC1-C-induced ISGs in HPAF-II and AsPC-1 cells, as well as PDAC tissues, are similar with those identified in *KRAS* mutant, ductal-derived PDAC tumors (9) and contribute to DNA damage resistance and cancer progression (25,47,48). The findings that MUC1-C activates the type I and II IFN pathways, which promote the CSC state (28,29,31), lend support for a potential model in which MUC1-C-induced ISGs contribute to NE dedifferentiation. However, further studies will be needed to determine whether (i) MUC1-C → IFN signaling drives stemness, as well as the NE phenotype and (ii) whether or not MUC1-C independently activates STAT1 and MYC (Figure 6G). In this regard, experiments that rescue STAT1, MYC, OSK, ASCL1, BRN2 and NOTCH1/2 expression will now be needed, at least in part, for defining the specific roles of these effectors. Another finding of potential importance is that HPAF-II and AsPC-1 cells, which are addicted to mutant KRAS for survival (21,22), are dependent on MUC1-C for self-renewal. These results suggested that, in addition to being necessary for activation of IFN signaling, MUC1-C may be essential for addiction to the *KRAS* mutant PDAC phenotype by driving other pathways linked to CSC progression.

The Yamanaka OSKM factors collectively dedifferentiate fibroblasts to induced pluripotent stem cells (iPSCs) in a manner that is potentiated by p53 and RB suppression (32). We found in HPAF-II and AsPC-1 cells, which harbor both p53 and RB mutations, that MUC1-C is necessary for OSKM expression, lending support to an unrecognized MUC1-C-driven pathway of pluripotency in PDAC cells. Viewed in this way, we found that MUC1-C associates with MYC and that silencing MUC1-C or MYC suppresses expression of the ASCL1 and BRN2 NE lineage-dictating TFs (Figure 6G). In addition, the MUC1-C → MYC pathway was necessary for expression of NOTCH1/2, indicating that MUC1-C integrates (i) lineage plasticity as evidenced by induction of the OSKM pluripotency factors, (ii) NE dedifferentiation as supported by upregulation of the ASCL1 and BRN2 neural TFs and (iii) stemness, which is driven by NOTCH1/2 signaling (Figure 6G). These findings collectively supported the notion that HPAF-II and AsPC-1 cells may have emerged as a result of intrinsic chronic activation of the IFN inflammatory pathways in association with driving dedifferentiation and plasticity from the ductal adenocarcinoma lineage specification. In concert with integration of these hallmark traits, MUC1-C was necessary for HPAF-II and AsPC-1 cell self-renewal capacity as evidenced by the findings that targeting MUC1-C genetically or pharmacologically inhibits tumorsphere formation, clonogenic survival and tumorigenicity. Previous studies demonstrated involvement of MUC1 in PDAC cell proliferation and invasion (49–51), whereas the present work has focused on PDAC lineage plasticity and NE dedifferentiation. Lineage tracing studies in the KCY mouse model demonstrated that acinar-to-ductal cell metaplasia occurs before PDAC development, leading to the conclusion that ductal-NE lineage plasticity drives PDAC progression, chemoresistance and poor clinical outcomes (36). These findings, taken together with activation of IFN pathways (9) and the present work, are collectively in agreement with a ductal cell of origin in PDAC progression.

Studies in castration-resistant prostate cancer cells have demonstrated that MUC1-C regulates lineage specification in the progression to NE prostate cancer (16). In that model, MUC1-C drives expression of the Yamanaka pluripotency factors and induces the ASCL1 and BRN2 neural TFs that dictate the NE lineage (16). These apparent parallels between MUC1-C-induced NE dedifferentiation in PDAC and NE prostate cancer tumors underscore a role for MUC1-C in driving lineage plasticity that contributes to NE cancer progression. Of importance in this regard, lineage plasticity of cancer cells is intimately associated with resistance to treatment with cytotoxic, targeted and immunotherapeutic agents (52). The progression of adenocarcinomas to NE carcinomas has been reported in colorectal tumors (53). In addition, colorectal NE carcinomas are genetically similar to colorectal adenocarcinomas, indicating a common cell of origin (54). Lineage plasticity of non-small cell lung cancers during the development of resistance to immunotherapy has also been associated with progression to small cell lung cancers with characteristics of NE dedifferentiation (55). Further study will be needed to determine if acquired resistance to PDAC treatment promotes progression to a NE phenotype. Nonetheless, our findings from analysis of the TCGA-PAAD dataset that MUC1 is associated with activation of the MYC and NOTCH signaling pathways and poor clinical outcomes hold potentially important implications in that MUC1-C could represent a potential target for the treatment of PDACs with lineage plasticity and NE dedifferentiation (Figure 6G). Notably in this regard, MUC1-C is a druggable target (10). Antibodies directed against the MUC1-C extracellular domain have been developed for CAR T cells that are entering the clinic in 2021, as well as antibody-drug conjugates (56) for targeting MUC1-C-expressing carcinomas. As an additional therapeutic approach, GO-203 targets the MUC1-C intracellular domain and is under preclinical development in a nanoparticle formulation for sustained delivery in the clinic (57). Based on the present work, these agents may be effective against pancreatic and other recalcitrant carcinomas with features of NE dedifferentiation.

## Supplementary Material

bgab097_suppl_Supplementary_Material

## Data Availability

The accession number for the RNA-seq data is GEO Submission GSE181961.

## Abbreviations

ASCL1: achaete-scute homolog 1
AURKA: aurora kinase A
CSC: cancer stem cell
DOX: doxycycline
ESC: embryonic stem cell
ISGs: IFN-stimulated genes
MUC1-C: MUC1 C-terminal subunit
NE: neuroendocrine
NOTCH: neurogenic locus notch homolog protein
OSKM: OCT4, SOX2, KLF4 and MYC
PanNEC: pancreatic neuroendocrine carcinoma
PDAC: pancreatic ductal adenocarcinoma
