# Correction to: MUC1-C dictates neuroendocrine lineage specification in pancreatic ductal adenocarcinomas

**DOI:** 10.1093/carcin/bgaf091

**Published:** 2025-12-23

This is a correction to Zhou Luan, Yoshihiro Morimoto, Atsushi Fushimi, Nami Yamashita, Wenhao Suo, Atrayee Bhattacharya, Masayuki Hagiwara, Caining Jin, Donald Kufe, MUC1-C dictates neuroendocrine lineage specification in pancreatic ductal adenocarcinomas, *Carcinogenesis*, Volume 43, Issue 1, January 2022, Pages 67–76, https://doi.org/10.1093/carcin/bgab097

After article publication, the authors notified the journal that there was an error in Figure 3G. The authors found that the same image for GAPDH was inadvertently included in Figures 3G and 5F. These concerns were also raised on PubPeer (see https://pubpeer.com/publications/EBD48944E5E6CB157BD7FD0F8C438C). This error does not affect the conclusions of the research.

The corrected version of Figure 3G is provided below.

**Figure:**
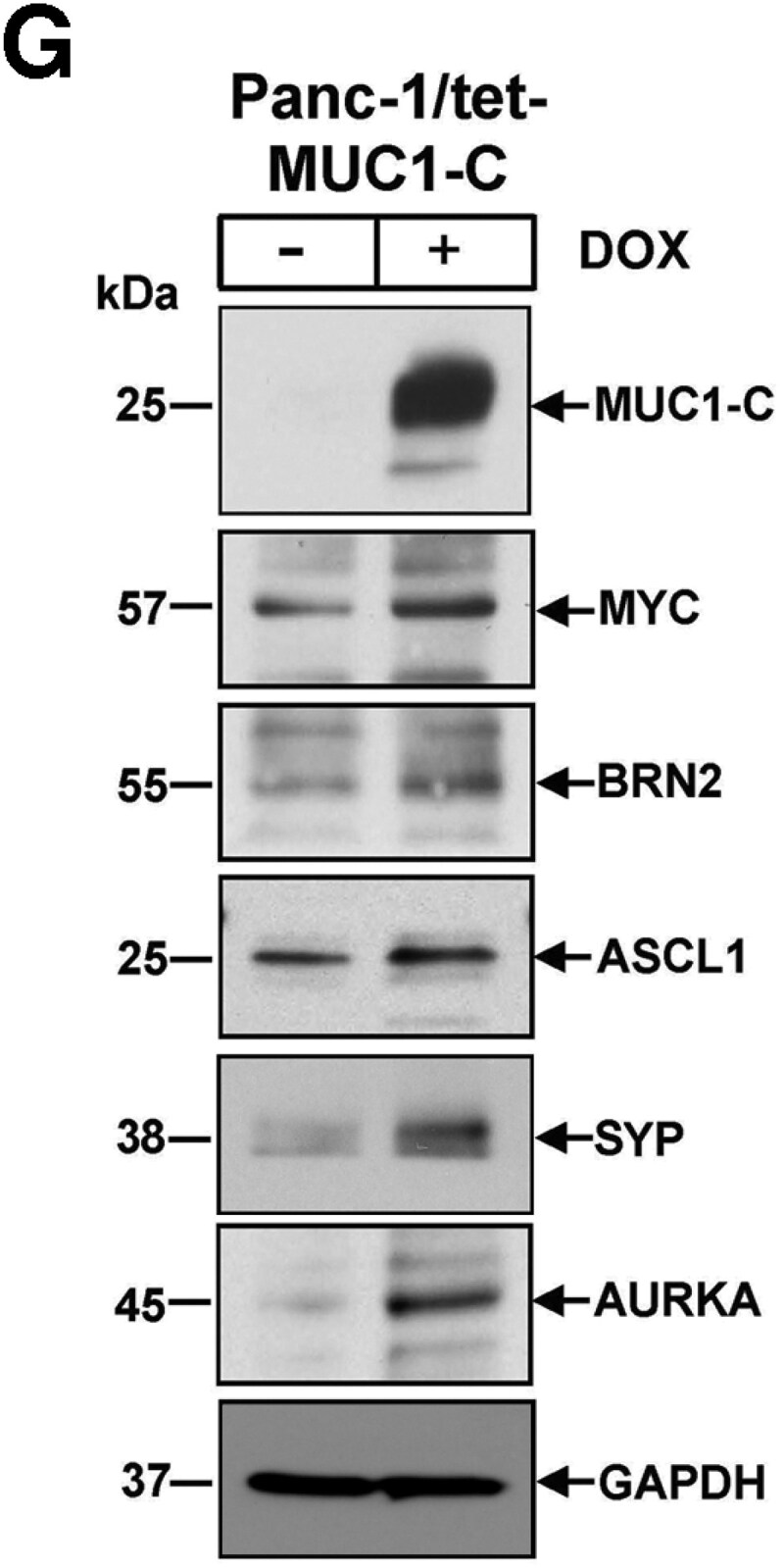


These details have been corrected only in this correction notice to preserve the published version of record.

