# Supplementary material for: MUC1-C dictates neuroendocrine lineage specification in pancreatic ductal adenocarcinomas
Source: Carcinogenesis. 2021 Oct 17;43(1):67–76. doi: 10.1093/carcin/bgab097 (PMC8832436; doi:10.1093/carcin/bgab097)
Supplement: bgab097_suppl_Supplementary_Material [file bgab097_suppl_supplementary_material.pdf]

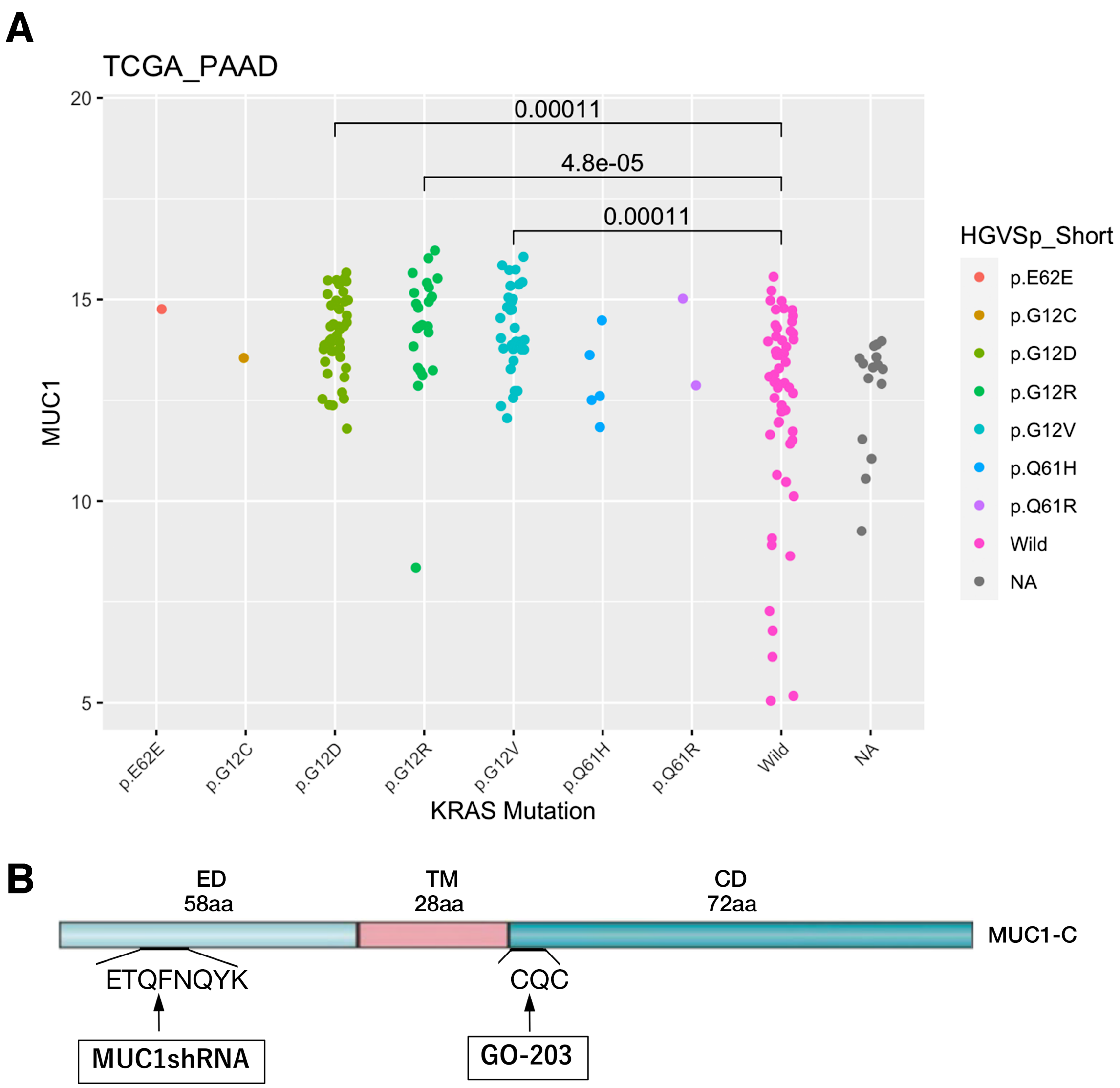


**Supplemental Figure S1. Associations of MUC1 expression with different *KRAS* mutations from analysis of the 163 PDAC tumors in the TCGA-PAAD/PDAC dataset. A.** MUC1 expression was significantly increased in tumors with *KRAS* G12D, G12R and G12V mutations as compared to *KRAS* wild-type tumors. Sample sizes for tumors with *KRAS* E62E, G12C, Q61H and Q61R mutations were not sufficient for making significant comparisons. NA: not available. **B.** Schematic representation of the MUC1-C subunit with the 58 aa extracellular domain, 28 aa transmembrane domain and the 72 aa cytoplasmic domain or tail. Highlighted are the aa sequences targeted by the MUC1shRNA and GO-203 inhibitor.

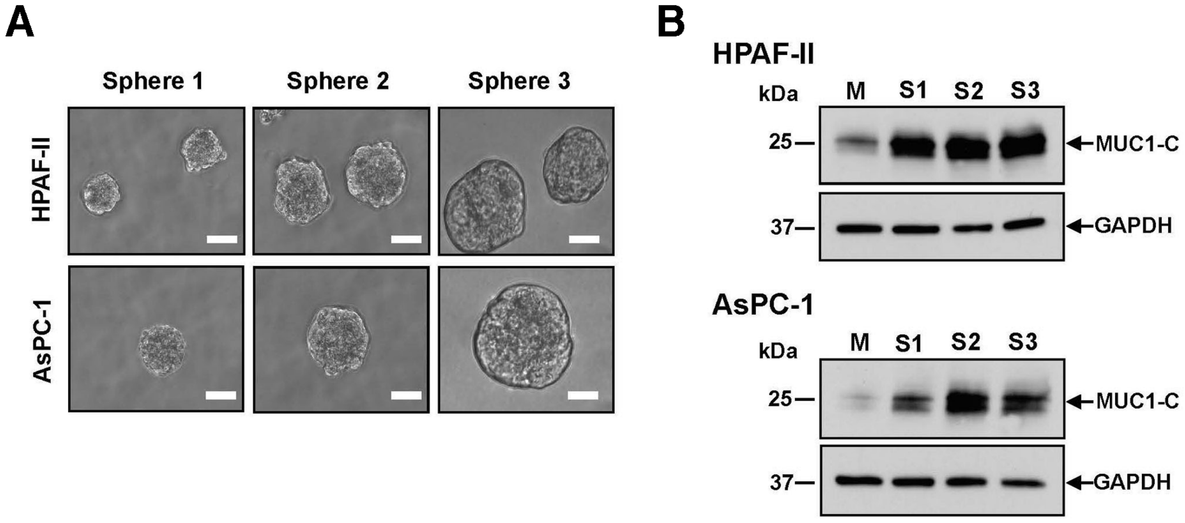


**Supplemental Figure S2. MUC1-C expression in HPAF-II and AsPC-1 cells grown as tumorspheres.** Schematic representation of the MUC1-C subunit with the 58 aa extracellular domain, 28 aa transmembrane domain and the 72 aa cytoplasmic domain or tail. Highlighted are the aa sequences targeted by the MUC1shRNA and GO-203 inhibitor. **A.** Representative images are shown for HPAF-II (upper) and AsPC-1 (lower) tumorspheres that were serially passaged for three generations (S1 to S3). Scale bar: 50 microns. **B.** Lysates from HPAF-II (upper) and AsPC-1 (lower) cells grown as adherent monolayers (M) and as tumorspheres (S1 to S3) were immunoblotted with antibodies against the indicated proteins.

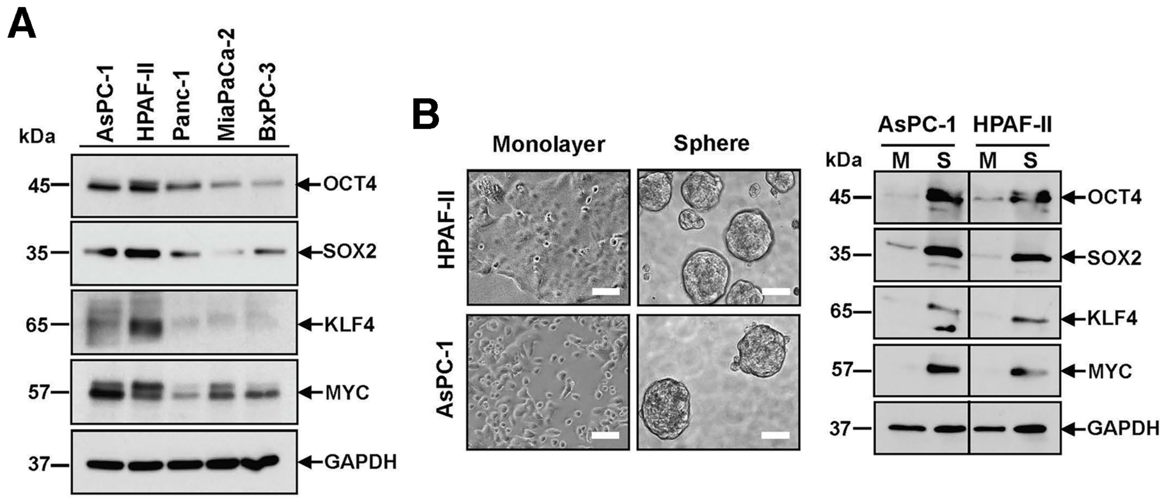


**Supplemental Figure S3. Expression of the Yamanaka OSKM factors in PDAC cells. A.** Lysates from the designated cell were immunoblotted with antibodies against the indicated proteins. **B.** Lysates from HPAF-II and AsPC-1 cells grown as monolayers (M) or spheres (S) (left) were immunoblotted with antibodies against the indicated proteins (right). As compared to panel A, intensity of the OSKM signals was decreased by shorter exposure time to demonstrate upregulation of their expression in spheres.

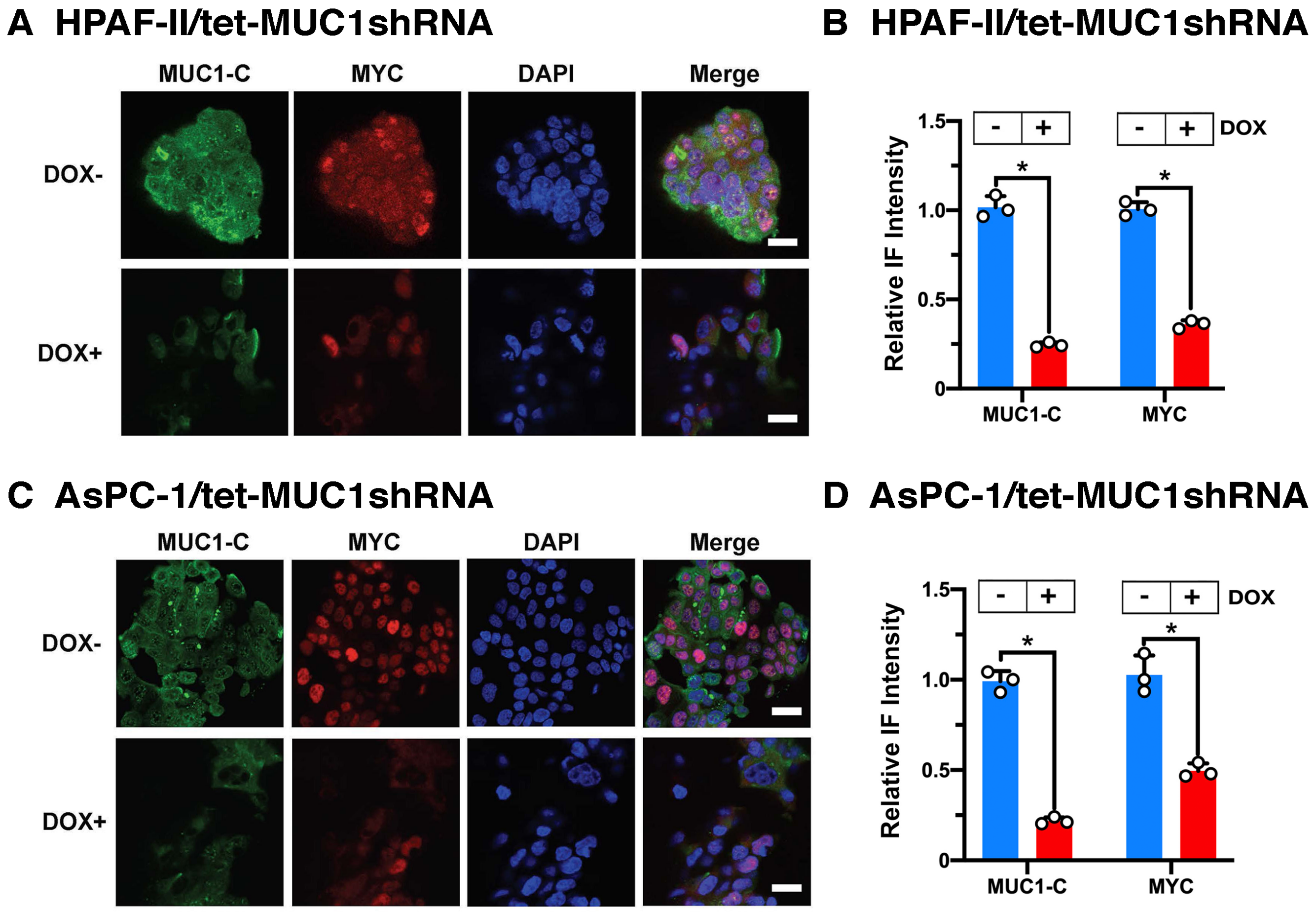


**Supplemental Figure S4. Silencing MUC1-C suppresses nuclear MUC1-C and MYC expression. A-D.** HPAF-II/tet-MUC1shRNA (**A,B**) and AsPC-1/tet-MUC1shRNA (**C,D**) cells treated with vehicle or DOX for 6 days were analyzed by confocal microscopy for MUC1-C and MYC expression. Nuclei were stained with DAPI. Representative images are shown for MUC1-C and MYC staining and for nuclear colocalization (merge). Scale bar: 50 microns. Relative immunofluorescence (IF) intensity was determined using ImageJ software. Results (mean±SD of three separate determinations) are expressed as the relative IF intensity compared to that obtained with the control cells (assigned a value of 1). The asterisk (*) denotes a significant difference from that obtained for control cells.

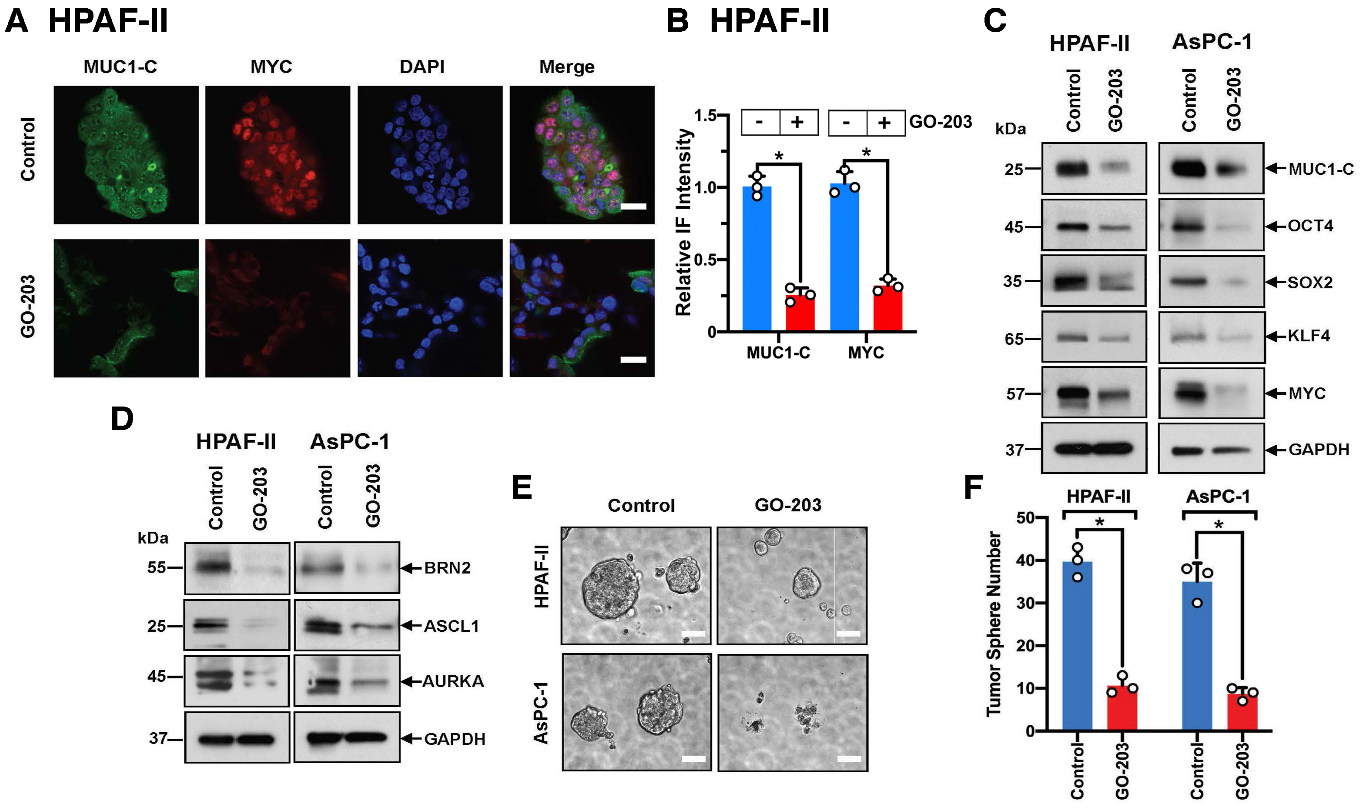


**Supplemental Figure S5. Targeting MUC1-C with GO-203 downregulates OSKM, NE markers and self-renewal. A.** HPAF-II cells left untreated or treated with 10 μM GO-203 for 48 hours were analyzed by confocal microscopy for MUC1-C and MYC expression. Nuclei were stained with DAPI. Representative images are shown for MUC1-C and MYC staining and for nuclear colocalization (merge). Scale bar: 50 microns. **B.** Relative immunofluorescence (IF) intensity was determined using ImageJ software. Results (mean±SD of three separate determinations) are expressed as the relative IF intensity compared to that obtained with the control cells (assigned a value of 1). The asterisk (*) denotes a significant difference from that obtained for control cells. **C and D.** Lysates from HPAF-II (left) and AsPC-1 (right) cells left untreated or treated with 10 μM GO-203 for 48 hours were immunoblotted with antibodies against the indicated proteins. **E.** Representative images are shown for HPAF-II (upper) and AsPC-1 (lower) cells suspended in tumorsphere medium in the absence and presence of 10 μM GO-203 for 72 hours (left). The results are expressed as tumorsphere number per well from 3 separate determinations of the indicated HPAF-II and AsPC-1 cells left untreated (blue bars) or treated with 10 μM GO-203 (red bars) for 72 hours (right). The asterisk (*) denotes a significant difference from that obtained for untreated control cells.

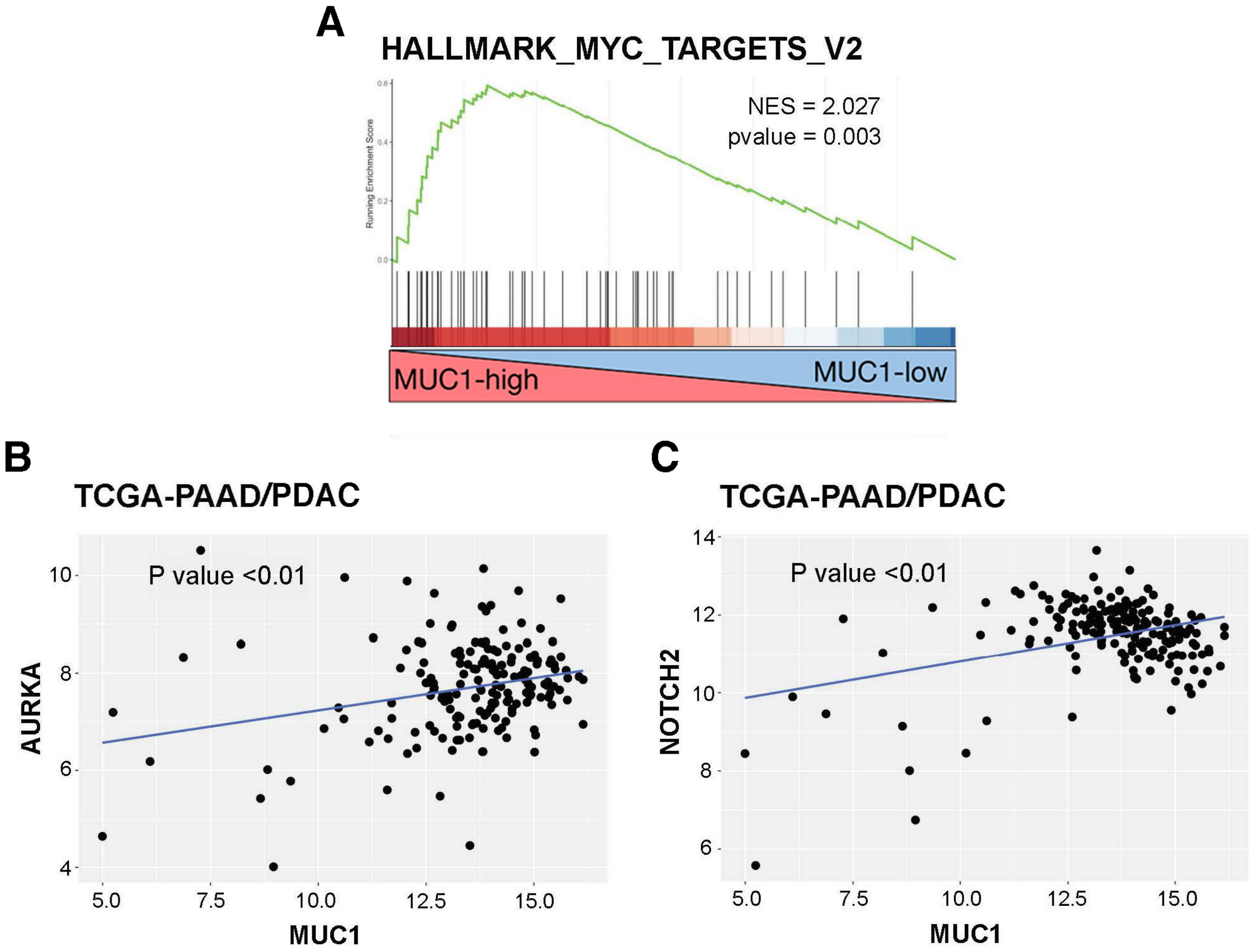


**Supplemental Figure S6. Analysis of MUC1 expression in PDAC tumors from the TCGA-PAAD/PDAC dataset. A.** Enrichment plot for the HALLMARK MYC TARGETS V2 pathway, comparing MUC1-high to MUC1-low PDAC tumors. **B and C.** Scatterplots showing correlations of MUC1 with AURKA (**B**) and NOTCH2 (**C**) expression.
